# Noninvasive Monitoring of Severe Pulmonary Artery Hypertension in Atrial Septal Defect Patients: Role of Serum Bilirubin Combined with Uric Acid

**DOI:** 10.31083/j.rcm2502050

**Published:** 2024-01-29

**Authors:** Feng Zhang, Dawei Lin, Qi Jin, Jianing Fan, Dandan Chen, Lihua Guan, Wenzhi Pan, Daxin Zhou

**Affiliations:** ^1^Department of Cardiology, Zhongshan Hospital, Fudan University, Shanghai Institute of Cardiovascular Diseases, National Clinical Research Center for Interventional Medicine, 200032 Shanghai, China; ^2^Department of Cardiology, Jinshan Hospital, Fudan University, 201508 Shanghai, China

**Keywords:** severe pulmonary artery hypertension, atrial septal defect, bilirubin, uric acid

## Abstract

**Background:**

Atrial septal defect (ASD) patients commonly experience severe pulmonary arterial hypertension (SPAH), which is frequently associated with a poor prognosis. While serum bilirubin levels, indicative of liver function, are known predictors of right heart failure (RHF), their potential to differentiate SPAH in ASD patients is yet to be ascertained. The purpose of this study was to discover the potential correlations between serum bilirubin levels and ASD patients with SPAH.

**Methods:**

In this cross-sectional study, 102 ASD patients admitted from December 2019 to November 2020 were enrolled and divided into two cohorts: those with SPAH and those without. Blood tests were conducted to measure serum direct bilirubin (DBIL), total bilirubin (TBIL), alanine aminotransferase (ALT), aspartate aminotransferase (AST), uric acid (UA) and N-terminal pro B-type natriuretic peptide (NT-proBNP). Additionally, all participants underwent transthoracic echocardiography, and invasive hemodynamic data were gathered through right heart catheterization.

**Results:**

ASD patients with SPAH exhibited significantly elevated serum DBIL (5.2 ± 3.0 vs. 2.4 ± 1.5 µmol/L, *p* < 0.001) and TBIL (24.6 ± 20.7 vs. 10.1 ± 4.8 µmol/L, *p* < 0.001) levels in comparison to those without SPAH. However, ALT and AST levels remained comparable between the cohorts. Additionally, the SPAH cohort displayed higher serum UA (403.5 ± 131.6 vs. 317.8 ± 67.9 µmol/L, *p* < 0.001) and NT-proBNP levels. Serum DBIL levels, when analyzed independently of other variables, correlated with an increased risk of mean pulmonary arterial pressure (mPAP) in ASD patients (β = 1.620, *p* = 0.010). A DBIL concentration of 2.15 mg/dL effectively differentiated ASD patients with SPAH from those without, with a sensitivity of 92.9% and a specificity of 51.4% (area under the curve [AUC]: 0.794, 95% confidence interval [CI]: 0.701–0.886, *p* < 0.001). Notably, the combination of DBIL and UA had a higher sensitivity of 92.9% and specificity of 71.6% (AUC: 0.874, 95% CI: 0.799–0.949, *p* < 0.001).

**Conclusions:**

Elevated serum DBIL and TBIL levels in ASD patients with SPAH were correlated with poor cardiac function and heightened pulmonary artery pressure. The combination of DBIL and UA has emerged as a strong noninvasive predictor for SPAH in ASD patients, presenting a potentially novel therapeutic biomarker.

## 1. Introduction

Atrial septal defect (ASD) is a frequently occurring congenital heart deficit. Between 6% and 35% of ASD patients develop pulmonary arterial hypertension (PAH), which can lead to increased mortality, diminished cardiac function, and atrial tachyarrhythmias [1, 2, 3, 4, 5, 6]. Severe pulmonary arterial hypertension (SPAH) in patients with ASD has a poor prognosis [7]. Currently, therapeutic strategies for ASD with SPAH are controversial. Our previous study demonstrated that mean pulmonary arterial pressure (mPAP) was a simple but powerful predictor of the benefits of ASD closure in these patients, with an optimal cut-off value of 35 mmHg and an area under the curve (AUC) of 0.919 [5].

Nevertheless, in ASD patients with SPAH, closure may not always decrease pulmonary artery pressure due to factors like vessel remodeling and decreased vascular compliance in ASD patients with SPAH [6]. Yong *et al*. [8] reported that after transcatheter ASD closure, most patients with severe PAH continue to have elevated pulmonary artery pressures, which might be due to irreversible vessel changes [7]. Therefore, it is important to identify SPAH in ASD patients to choose the best timing for ASD occlusion. While right heart catheterization has long been the gold standard for determining SPAH, the procedure is invasive. There is a growing emphasis on biomarker studies to prompt an earlier initiation of more aggressive therapies. These biomarkers may be used for identifying potential SPAH in ASD patients.

Bilirubin is a promising biomarker for assessing patients within this population. It is a metabolic byproduct of hemoglobin breakdown that also acts as an endogenous antioxidant molecule [9]. Total bilirubin (TBIL) is well established prognostic factor in heart failure [10] and PAH [8]. However, most prior PAH studies focused on patients with predominantly idiopathic or connective tissue disease-related cases. Few studies have focused solely on ASD-associated PAH, which has distinct pulmonary hemodynamics and pathophysiology. Since the role of bilirubin in ASD-SPAH remains unknown, this study was undertaken to investigate the role of serum bilirubin to assess its significance in ASD patients, especially in those with SPAH.

## 2. Methods

### 2.1 Study Populations

This cross-sectional study included 102 patients with ASD admitted to Zhongshan Hospital, Fudan University, from December 2019 to November 2022. The diagnosis of PAH was based on right-heart catheterization values [11]: mPAP ≥20 mmHg, pulmonary arterial wedge pressure (PAWP) ≤15 mmHg, and pulmonary vascular resistance (PVR) >3 Wood units. Patients were categorized based on catheter mPAP as: normal (≤20 mmHg), mild (20–35 mmHg), moderate (35–45 mmHg), or severe PAH (>45 mmHg) [12]. To identify patients with SPAH, these individuals were divided into two groups (ASD with SPAH and ASD without SPAH) according to a mPAP >45 mmHg. Exclusion criteria: (1) patients with patent foramen ovale, significant pulmonary stenosis, or Ebstein’s anomaly; (2) patients with other identifiable causes for precapillary pulmonary hypertension, such as chronic thromboembolic pulmonary hypertension; (3) lung transplantation before ASD closure; (4) patients who have already been treated with specific PAH agents; (5) diseases resulting in hyperbilirubinemia, such as chronic hepatitis, malignant tumors, administration of hepatotoxic drugs, alcohol dependence, hemolysis and hematoma reabsorption, schistosomiasis, and biliary system diseases. A detailed medical history and abdominal ultrasonography were performed to exclude chronic liver disease. Urine samples were performed to screen for hemolysis or hematoma absorption.

Baseline characters and clinical data were collected from electronic medical records. TTE was performed using Vivid 7GE (G.E. Healthcare, Chicago, IL, USA) by an experienced sonographer, and echocardiographic parameters including left ventricular ejection fraction (LVEF), left atrial diameter (LAD), and left ventricular end-diastolic (LVED) were validated by two cardiology consultants. The diameter, type, and size of the defect and the anatomy of the ASD were examined by cardiology consultants. This study was performed in compliance with the Helsinki Declaration and was approved by the Ethics Committee. Written informed consent was obtained from all participants.

### 2.2 Blood Collection and Biochemical Measurements 

The peripheral venous blood samples were obtained with the consent of the patients. Serum TBIL, direct bilirubin (DBIL), uric acid (UA), aspartate transaminase (ASL), alanine transaminase (ALT), and N-terminal pro B-type natriuretic peptide (NT-proBNP) were measured just before right heart catheterization at the Central Clinical Laboratory of our Hospital. The normal value of TBIL is 1.71–17.1 µmol/L, and DBIL 1.71–7.0 µmol/L in our lab.

### 2.3 Right Heart Catheterization

Right heart catheterization was employed by cardiologists to evaluate PAH severity. A multipurpose catheter was inserted into the right atrium via the inferior vena cava and placed in the pulmonary veins across the ASD. A Swan-Ganz balloon-tipped catheter was placed in the pulmonary arteries through the right atrium and right ventricle. Hemodynamic parameters such as pulmonary arterial systolic pressure (PASP) and mPAP were then evaluated.

### 2.4 Statistical Analysis

Continuous variables with a normal distribution were presented as mean ± SD while non-normally distributed data were presented as median (25th–75th percentile). Categorical data were described as percentages. The Unpaired Student’s *t* test and the χ^2^ test were used to analyze the statistical differences between the two groups. Correlations between continuous data (for normally distributed data) were analyzed with Pearson correlation tests. Single-variate and multi-variate linear regression analyses were performed to evaluate the degree of correlation between plasma biomarkers and clinical indicators. Sensitivity, specificity, as well as areas under the curve (AUC) were obtained using receiver operating characteristic (ROC) curve analysis. Results were considered statistically significant for *p* values < 0.05. All statistical analyses were performed using Stata 15.1 (StataCorp LP, College Station, TX, USA) or SPSS 26.0 (IBM Corp., Armonk, NY, USA).

## 3. Results

### 3.1 Basic Characteristics

In total, 102 patients were included in the study, 28 were in the ASD with SPAH cohort and 74 were in ASD without SPAH cohort. Their baseline characters, clinical, biochemical, hemodynamic, and echocardiographic data are presented in Table 1. There were no statistically significant differences in terms of mean age, sex, LAD, and LVEF between the two cohorts. However, the ASD with SPAH cohort had a significantly larger defect size (26.0 ± 9.9 vs. 16.4 ± 7.5 mm, *p* < 0.001), right ventricle diameter (RVD) (49.3 ± 5.1 vs. 33.1 ± 9.1 mm, *p* < 0.001), more severe tricuspid regurgitation (TR, 53.6% vs.14.9%, *p* < 0.001), lower LVED (37.8 ± 7.7 vs. 43.5 ± 5.3 mm, *p* < 0.001), and tricuspid annular plane systolic excursion (TAPSE) (14.9 ± 1.9 vs. 19.6 ± 3.0 mm, *p* < 0.001) when compared with the ASD without SPAH cohort.

**Table 1.** **Baseline and clinical characteristics of ASD patients with or without SPAH**.

Variables		ASD without SPAH	ASD with SPAH	*p* values
		n = 74	n = 28	
Age, years		42.4 ± 15.9	45.5 ± 16.6	0.38
Male (%)		25 (33.8%)	6 (21.4%)	0.23
TTE and RHC				
	ASD diameter, mm	16.4 ± 7.5	26.0 ± 9.9	< **0.001**
	PASP, mmHg	33.0 ± 11.4	83.9 ± 16.6	< **0.001**
	mPAP, mmHg	24.3 ± 10.4	50.4 ± 4.4	< **0.001**
	PADP, mmHg	9.1 ± 4.5	23.6 ± 6.9	< **0.001**
	TAPSE, mm	19.6 ± 3.0	14.9 ± 1.9	< **0.001**
	LVEF, %	65.4 ± 3.6	64.8 ± 4.2	0.45
	LAD, mm	37.1 ± 5.4	38.6 ± 7.0	0.25
	LVED, mm	43.5 ± 5.3	37.8 ± 7.7	< **0.001**
	RVD, mm	33.1 ± 9.1	49.3 ± 5.1	< **0.001**
	TR > Grade 1	11 (14.9%)	15 (53.6%)	< **0.001**
Blood Examination				
	DBIL, µmol/L	2.4 ± 1.5	5.2 ± 3.0	< **0.001**
	TBIL, µmol/L	10.1 ± 4.8	24.6 ± 20.7	< **0.001**
	DBIL/TBIL	0.2 ± 0.1	0.3 ± 0.1	0.52
	ALT, U/L	20.7 ± 14.4	18.1 ± 6.4	0.37
	AST, U/L	19.5 ± 5.7	20.6 ± 9.1	0.51
	UA, µmol/L	317.8 ± 67.9	403.5 ± 131.6	< **0.001**
	NT-proBNP, pg/mL	52 (33.3, 119.3)	525 (129.3, 626)	< **0.001**

Data are presented as mean ± standard deviation or number (%) of patients. Non-normal distribution data are shown as median (25th–75th percentile). Abbreviation: ASD, atrial septal defect; SPAH, severe pulmonary arterial hypertension; TTE, transthoracic echocardiography; RHC, right heart catheterization; PASP, pulmonary arterial systolic pressure; PADP, pulmonary arterial diastolic pressure; mPAP, mean pulmonary arterial pressure; TAPSE, tricuspid annular plane systolic excursion; LVEF, left ventricular ejection fraction; LAD, left atrial diameter; LVED, left ventricular end diastolic; RVD, right ventricle diameter; TR, tricuspid regurgitation; DBIL, direct bilirubin; TBIL, total bilirubin; ALT, alanine aminotransferase; AST, aspartate aminotransferase; UA, uric acid; NT-proBNP, N-terminal pro B-type natriuretic peptide. Note: *p* < 0.05 was indicated in bold.

### 3.2 Serum Biomarkers between Groups

**Fig. 1.**
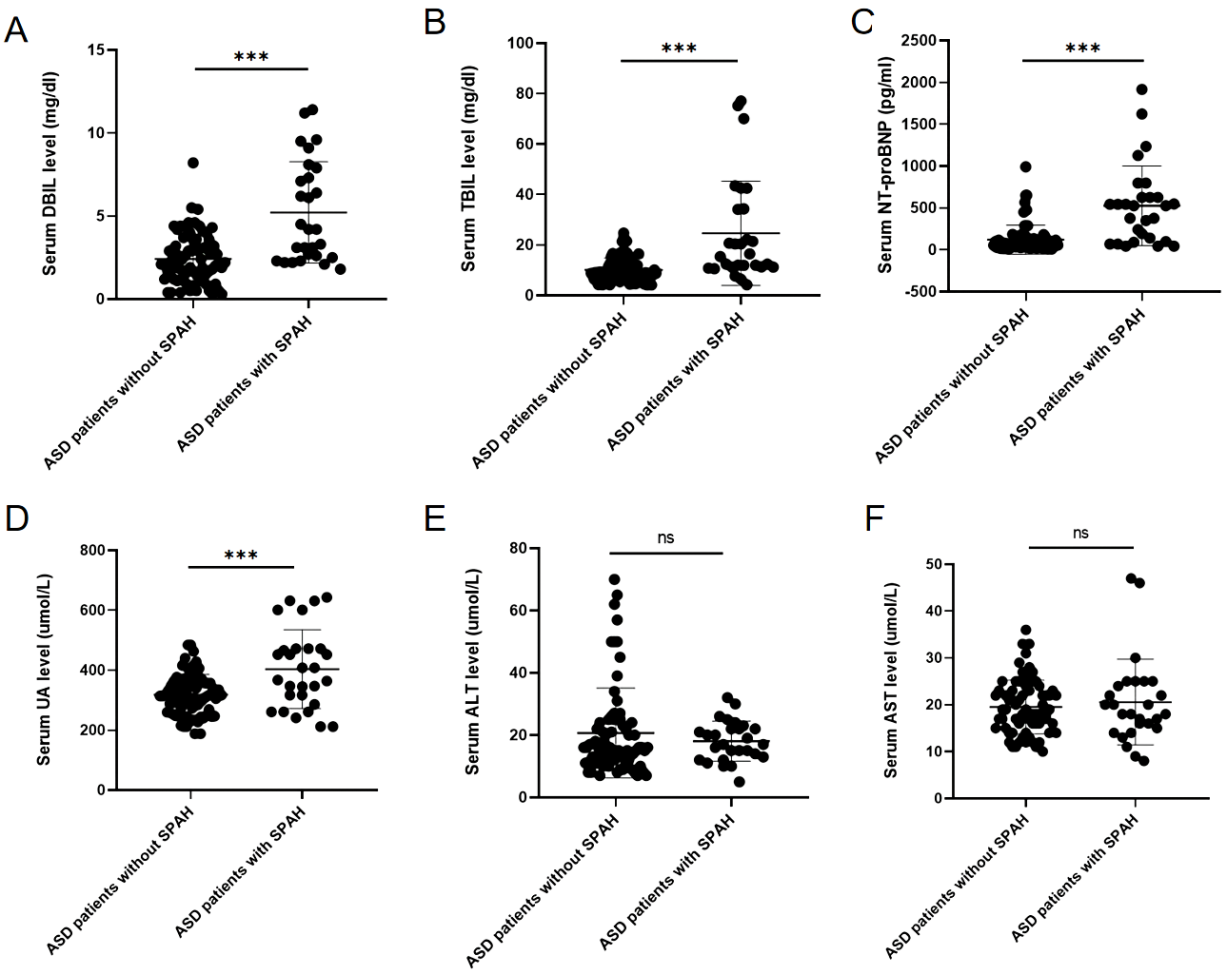
**Serum biomarkers levels in ASD patients with or without SPAH**. (A–F) demonstrated the serum DBIL, TBIL, NT-proBNP, UA, ALT, and AST level in ASD patients with and without SPAH groups respectively (*** means *p* < 0.001, ns means *p* > 0.5). Abbreviation: ASD, atrial septal defect; SPAH, severe pulmonary artery hypertension; DBIL, direct bilirubin; TBIL, total bilirubin; ALT, alanine aminotransferase; AST, aspartate aminotransferase; UA, uric acid; NT-proBNP, N-terminal pro B-type natriuretic peptide.

Table 1 indicates that ASD patients with SPAH had significantly higher serum levels of DBIL (5.2 ± 3.0 vs. 2.4 ± 1.5 µmol/L, *p* < 0.001), TBIL (24.6 ± 20.7 vs. 10.1 ± 4.8 µmol/L, *p* < 0.001), and UA (403.5 ± 131.6 vs. 317.8 ± 67.9 µmol/L, *p* < 0.001) compared to those without SPAH. In addition, ASD patients with SPAH had higher serum NT-proBNP levels (525 [129.3, 626] vs. 52 [33.3, 110.3] pg/mL, *p* < 0.001). However, there were no significant differences in the DBIL/TBIL ratio, ALT, or aspartate aminotransferase (AST) between the cohorts (Fig. 1). **Supplementary Table 1** provides a comparative analysis of blood biomarkers in the ASD cohorts stratified by mean pulmonary arterial pressure levels. The levels of DBIL, TBIL, UA, and NT-proBNP progressively increased with increasing mPAP levels, suggesting a possible relationship between these variables and disease severity.

### 3.3 Correlations between Parameters

**Fig. 2.**
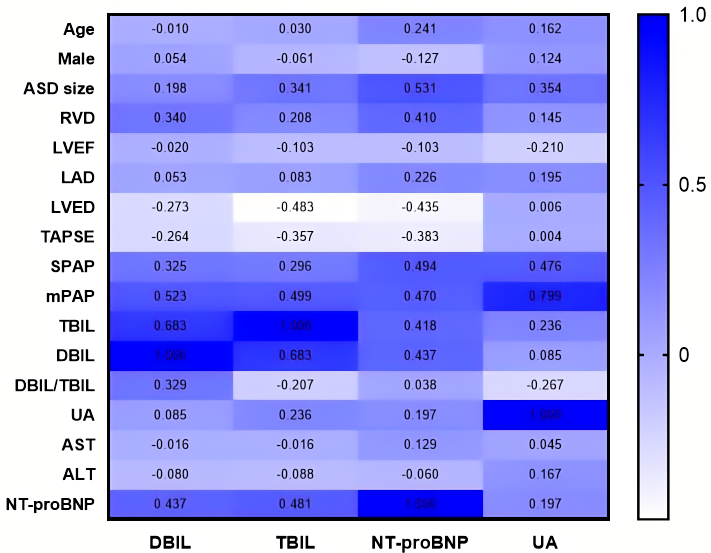
**Correlations of serum biomarkers with various parameters**. Abbreviation: ASD, atrial septal defect; RVD, right ventricle diameter; mPAP, mean pulmonary arterial pressure; LVEF, left ventricular ejection fraction; LAD, left atrial diameter; LVED, left ventricular end diastolic; DBIL, direct bilirubin; TBIL, total bilirubin; UA, uric acid; NT-proBNP, N-terminal pro B-type natriuretic peptide; TAPSE, tricuspid annular plane systolic excursion; ALT, alanine aminotransferase; AST, aspartate aminotransferase; SPAP, pulmonary artery systolic pressure.

Using the Pearson correlation, relationships between variables were analyzed. These variables include baseline characters (age, male), biochemical and hemodynamic variables (DBIL, TBIL, DBIL/TBIL, NT-proBNP, UA, AST and ALT), echocardiographic parameters (ASD diameter, RVD, LVEF, LAD, TAPSE) and right heart catheterization (pulmonary artery systolic pressure [sPAP], mPAP). Serum DBIL levels had a positive correlation with multiple cardiac ultrasound, right heart catheterisation, and blood biochemistry indicators. While serum TBIL showed positive correlations with some factors (ASD diameter, RVD, sPAP, mPAP, DBIL, UA, and NT-proBNP), there were negative correlations with others (LVED, TAPSE, and DBIL/TBIL ratio). Serum NT-proBNP also had many significant correlations (age, ASD diameter, RVD, LAD, LVED, TAPSE, sPAP, mPAP, TBIL, DBIL, and UA). Finally, serum UA was also correlated with various factors (ASD diameter, LVEF, LAD, sPAP, mPAP, TBIL, DBIL/TBIL ratio, and NT-proBNP). More detailed statistics, including r values and levels of significance can be found in Table 2 and Fig. 2.

**Table 2.** **Correlations of DBIL and TBIL with various parameters in ASD patients**.

Variables	DBIL		TBIL		NT-proBNP		UA	
	r	*p*	r	*p*	r	*p*	r	*p*
Age	–0.010	0.924	0.030	0.763	0.241	**0.015**	0.162	0.103
Male	0.054	0.102	–0.061	0.544	–0.127	0.203	0.124	0.215
ASD diameter	0.198	**0.047**	0.341	< **0.001**	0.531	< **0.001**	0.354	< **0.001**
RVD	0.340	< **0.001**	0.208	**0.036**	0.410	< **0.001**	0.145	0.102
LVEF	–0.020	0.840	–0.103	0.192	–0.103	0.303	–0.210	**0.034**
LAD	0.053	0.594	0.083	0.408	0.226	**0.022**	0.195	0.050
LVED	–0.273	< **0.001**	–0.483	< **0.001**	–0.435	< **0.001**	0.006	0.952
TAPSE	–0.264	**0.007**	–0.357	< **0.001**	–0.383	< **0.001**	0.004	0.102
sPAP	0.325	< **0.001**	0.296	**0.003**	0.494	< **0.001**	0.467	< **0.001**
mPAP	0.523	< **0.001**	0.499	< **0.001**	0.470	< **0.001**	0.799	< **0.001**
TBIL	0.683	< **0.001**	-	-	0.418	< **0.001**	0.236	**0.017**
DBIL	-	-	0.683	< **0.001**	0.437	< **0.001**	0.085	0.397
DBIL/TBIL	0.329	< **0.001**	–0.207	**0.037**	0.038	0.703	–0.267	**0.007**
UA	0.085	0.397	0.236	**0.017**	0.197	**0.047**	-	-
AST	–0.016	0.492	–0.016	0.878	0.129	0.200	0.045	0.658
ALT	–0.080	0.428	–0.088	0.382	–0.060	0.551	0.167	0.100
NT-proBNP	0.437	< **0.001**	0.418	< **0.001**	-	-	0.197	**0.047**

Abbreviation: ASD, atrial septal defect; RVD, right ventricle diameter; mPAP, mean pulmonary arterial pressure; LVEF, left ventricular ejection fraction; LAD, left atrial diameter; LVED, left ventricular end diastolic; DBIL, direct bilirubin; TBIL, total bilirubin; UA, uric acid; NT-proBNP, N-terminal pro B-type natriuretic peptide; TAPSE, tricuspid annular plane systolic excursion; ALT, alanine aminotransferase; AST, aspartate aminotransferase; sPAP, pulmonary artery systolic pressure. Note: *p* < 0.05 was indicated in bold.

### 3.4 Association of Serum Biomarkers and mPAP among ASD Patients

To explore the association between various parameters and PASP in ASD patients, linear regression analyses were performed (Table 3). Single-variable linear regression showed that ASD diameter, TBIL, DBIL, RVD, TAPSE, NT-proBNP and UA were significantly associated with increased mPAP among ASD patients with SPAH. Further multivariate linear regression analysis demonstrated that serum DBIL (β = 1.552, *p* = 0.015) independently correlated with PASP, regardless of other variables including ASD diameter, RVD, TAPSE, TBIL, NT-proBNP, and UA. Similarly, UA levels (β = 0.030, *p* = 0.007) were independently associated with mPAP in ASD patients with SPAH, when controlled for other parameters such as ASD diameter, RVD, TAPSE, TBIL, NT-proBNP, and DBIL.

**Table 3.** **Single-variable and multi-variable linear regression analyses of association between variables and mPAP**.

Variable	Single-variable		Multi-variable	
	β	*p*	β	*p*
ASD diameter	0.679	< **0.001**	0.125	0.358
RVD	0.775	< **0.001**	0.370	**0.001**
TAPSE	–2.503	< **0.001**	–1.083	**0.003**
TBIL	0.016	< **0.001**	0.106	0.343
DBIL	3.258	< **0.001**	1.552	**0.015**
DBIL/TBIL	–3.278	0.796	-	-
ALT	–0.152	0.192	-	-
AST	0.027	0.902	-	-
NT-proBNP	0.021	< **0.001**	0.001	0.690
UA	0.059	< **0.001**	0.030	**0.007**

Abbreviation: mPAP, mean pulmonary arterial pressure; ASD, atrial septal defect; TAPSE, tricuspid annular plane systolic excursion; RVD, right ventricle diameter; DBIL, direct bilirubin; TBIL, total bilirubin; ALT, alanine aminotransferase; AST, aspartate aminotransferase; UA, uric acid; NT-proBNP, N-terminal pro B-type natriuretic peptide. Note: *p* < 0.05 was indicated in bold.

### 3.5 ROC Curve Analyses of Serum DBIL Level for Predicting SPAH in ASD Patients

**Fig. 3.**
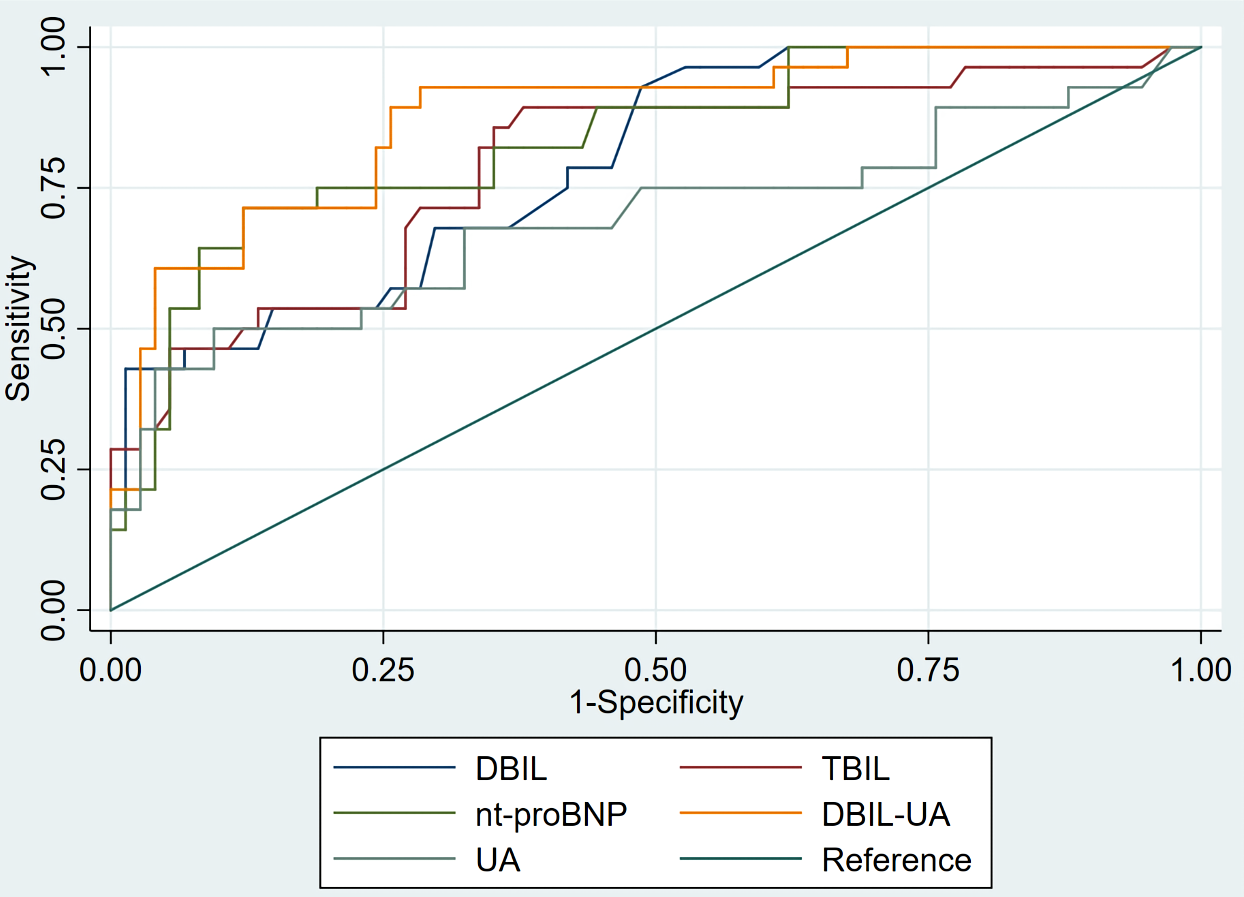
**ROC analyses of serum biomarkers predicting ASD patients with SPAH**. Abbreviation: ROC, receiver operating characteristic; ASD, atrial septal defect; SPAH, severe pulmonary artery hypertension; DBIL, direct bilirubin; TBIL, total bilirubin; UA, uric acid; NT-proBNP, N-terminal pro B-type natriuretic peptide.

Table 3, Fig. 3 and **Supplementary Table 2** outline distinct discriminative values. A DBIL level of 2.15 mg/dL is effective in distinguishing ASD with SPAH patients from those without SPAH, achieving a sensitivity of 92.9% and a specificity of 51.4% (AUC: 0.794, 95% confidence interval [CI]: 0.701–0.886, *p* < 0.001). Similarly, a TBIL level of 10.35 mg/dL offers a sensitivity of 89.3% and a specificity of 62.2% in differentiating between the two groups (AUC: 0.788, 95% CI: 0.685–0.890, *p* < 0.001). Furthermore, UA levels exceeding 407 µmol/L distinguish SPAH patients from the general ASD population with a sensitivity of 50.0% and a specificity of 90.5% (AUC: 0.693, 95% CI: 0.563–0.824, *p* = 0.003). Additionally, an NT-proBNP cutoff value of 187.5 pg/mL provides a means to differentiate SPAH patients from ASD patients with a sensitivity of 71.4% and a specificity of 87.5%. (AUC: 0.836, 95% CI: 0.748–0.923, *p* < 0.001). More importantly, the combination of DBIL and UA metrics result in an enhanced sensitivity of 92.9% and a specificity of 71.6% (AUC: 0.874, 95% CI: 0.799–0.949, *p* < 0.001). As shown in **Supplementary Figs. 1,2** further highlight that the sensitivity of combined DBIL and UA surpasses that of RVD and TAPSE in distinguishing between the ASD groups.

## 4. Discussion

This study demonstrates that elevated bilirubin levels (both DBIL and TBIL) are present in patients with both ASD and SPAH. Both DBIL and TBIL levels correlated with the cardiac function markers NT-proBNP, RVD, TAPSE, LVED, PASP and mPAP. Moreover, DBIL alone or in combination with UA can differentiate between ASD patients with SPAH and without SPAH with relatively high sensitivity and specificity.

Our study showed serum TBIL and DBIL were increased in ASD patients with SPAH, but without any concurrent increase in AST or ALT. The mechanisms causing elevated serum levels of TBIL and DBIL in ASD patients with SPAH are still unclear. One hypothesis suggests that for ASD patients, the elevated mPAP may increase the right atrial and ventricular load, which is expected to increase the risk of right heart failure (RHF). This RHF condition could manifest as liver dysfunction, potentially due to hepatic stasis and reduced perfusion. Changes in the right heart hemodynamic may be due to elevated central venous pressure (CVP) via the hepatic vein into the small hepatic veins [13]. This impairment can further hinder the delivery of oxygen and nutrients to hepatocytes, resulting in the expansion of sinusoidal fenestrations [13]. The resulting cholestatic and hypoxic hepatic injury caused by elevated CVP may contribute to an increase of bilirubin. In fact, DBIL has been identified as a critical marker of elevated CVP in other studies [10, 14, 15]. Furthermore, other researchers have highlighted the positive correlation between serum bilirubin with right atrial pressure and the severity of tricuspid regurgitation [16]. In our study, the damage to hepatocytes was limited, as indicated by the absence of statistical differences in ALT and AST between ASD with or without the SPAH. However, the liver biomarkers DBIL and TBIL were reflective of right heart function and were associated with the risk of SPAH in ASD patients.

Some basic researchers have sought to explore the relationship between bilirubin and PAH. Bilirubin is a tetrapyrrole pigment in blood that has both direct and indirect forms, and is known for its antioxidant and anti-inflammatory activity [17, 18, 19]. Physiological concentrations of bilirubin inhibit nuclear factor kappa-B (NF-κB) activation and inflammasome, contributing to its anti-inflammatory effects [17]. Thus, specific quantities of bilirubin may serve as a mitochondria-targeting agent for treating relevant diseases [20]. Prior studies have shown that bilirubin reductase attenuates the effects of hypoxia on apoptosis in pulmonary artery smooth muscle cells by modulating the bilirubin-mediated (extracellular signal-regulated kinase 1/2) ERK1/2 pathway [21]. Furthermore, it has been shown that oxidative stress serves as a critical etiological factor in the progression of PAH [22, 23, 24]. A study by Curjuric *et al*. [25] examined the influences of air pollution on adult lung disease, identifying a protective effect of bilirubin on lung function, emphasizing its potential preventive and curative significance. Therefore, increased bilirubin may have a protective effect on ASD patients with SPAH.

Our study demonstrated that the serum DBIL and TBIL levels can predict SPAH in ASD patients, with DBIL demonstrating a sensitivity of 92.9% and a specificity of 51.4%, while TBIL exhibited a sensitivity of 89.3% and a specificity of 62.2%. Echoing our observations, Xu *et al*. [26] showed that abnormally elevated DBIL was independently linked to all-cause mortality among idiopathic PAH patients, and treating PAH survivors significantly decreased serum DBIL levels. In contrast, almost no decrease in serum DBIL was found in non-survivors [27]. In addition, hyper-bilirubinemia (TBIL) was associated with advanced RHF, which then markedly reduced survival in patients with PAH [24]. Gong *et al*. [28] found that elevated serum bilirubin and reduced six-minute walk distance (6MWD) was identified as a predictor of adverse outcomes in patients with chronic thromboembolic pulmonary hypertension. While our study did not evaluate the prognostic implications for ASD patients with abnormal DBIL and TBIL levels, we observed that patients with SPAH presented more severe structural and hemodynamic symptoms compared to their non-SPAH counterparts. Therefore, our future research will focus on defining the role of DBIL and TBIL in predicting the prognosis of ASD patients with SPAH.

We observed that ASD patients with SPAH had significantly higher UA levels when compared to those without SPAH. Supporting this, Yan *et al*. [29] found that baseline hyperuricemia and high variability in serum UA were associated with higher 5-year mortality in patients with idiopathic PAH (IPAH). Savale *et al*. [30] suggests that UA levels can partly reflect the severity of PAH, with higher concentrations of UA promoting mild proliferation of pulmonary artery smooth muscle cells in patients with idiopathic PAH and in rat models. In our studies, DBIL levels combined with UA had a sensitivity (92.9%) and specificity (71.6%) to discriminated ASD individuals with SPAH. Therefore, UA combined with DBIL might better predict poor prognosis in these patients.

### Limitations

Our study has several limitations. Firstly, 6MWD was not analyzed in regression analysis due to incomplete data, preventing us from supplementing the study with 6MWD results. Secondly, the organizational roles of DBIL and TBIL in ASD patients with SPAH have not been explored in our study. Attempts to solve this problem using both animal and cellular experiments are underway. Thirdly, long-term follow-up should be conducted to evaluate whether DBIL and TBIL could predict the prognosis of ASD patients suffering from SPAH. In conclusion, given our current sample size, it’s challenging to rule out potential discrepancies. Further extensive, large-scale studies are needed to validate our findings.

## 5. Conclusions

Overall, we found that elevated serum DBIL and TBIL levels in ASD patients with SPAH are correlated with prognostic clinical markers. Utilizing DBIL combined with UA may serve as a safe, cost-effective, and powerful predictor of SPAH in patients with ASD, potentially introducing a novel therapeutic biomarker.

## Data Availability

The datasets generated and/or analyzed during the current study are not publicly available due to local rules national laws but are available from the corresponding author on reasonable request.

## Supplementary Material

Supplementary material associated with this article can be found, in the online version, at https://doi.org/10.31083/j.rcm2502050.

## References

[1] Engelfriet PM, Duffels MGJ, Möller T, Boersma E, Tijssen JGP, Thaulow E (2007). Pulmonary arterial hypertension in adults born with a heart septal defect: the Euro Heart Survey on adult congenital heart disease. *Heart*.

[2] Vogel M, Berger F, Kramer A, Alexi-Meshkishvili V, Lange PE (1999). Incidence of secondary pulmonary hypertension in adults with atrial septal or sinus venosus defects. *Heart*.

[3] Steele PM, Fuster V, Cohen M, Ritter DG, McGoon DC (1987). Isolated atrial septal defect with pulmonary vascular obstructive disease–long-term follow-up and prediction of outcome after surgical correction. *Circulation*.

[4] Craig RJ, Selzer A (1968). Natural history and prognosis of atrial septal defect. *Circulation*.

[5] Lilyasari O, Istisakinah R, Ariani R, Rahmat B, Liastuti LD, Kurniawati Y (2022). Operability of atrial septal defect with borderline pulmonary vascular resistance index: A study in developing country. *Frontiers in Surgery*.

[6] Dinarti LK, Hartopo AB, Kusuma AD, Satwiko MG, Hadwiono MR, Pradana AD (2020). The COngenital HeARt Disease in adult and Pulmonary Hypertension (COHARD-PH) registry: a descriptive study from single-center hospital registry of adult congenital heart disease and pulmonary hypertension in Indonesia. *BMC Cardiovascular Disorders*.

[7] Ranard LS, Mallah WE, Awerbach JD, Abernethy A, Halane M, Qureshi AM (2019). Impact of Pulmonary Hypertension on Survival Following Device Closure of Atrial Septal Defects. *The American Journal of Cardiology*.

[8] Yong G, Khairy P, De Guise P, Dore A, Marcotte F, Mercier LA (2009). Pulmonary arterial hypertension in patients with transcatheter closure of secundum atrial septal defects: a longitudinal study. *Circulation. Cardiovascular Interventions*.

[9] Pan W, Zhang Y, Guan L, Zhang X, Zhang L, Yang L (2020). Usefulness of mean pulmonary artery pressure for predicting outcomes of transcatheter closure of atrial septal defect with pulmonary arterial hypertension. *EuroIntervention*.

[10] Muneuchi J, Ochiai Y, Masaki N, Okada S, Iida C, Sugitani Y (2019). Pulmonary arterial compliance is a useful predictor of pulmonary vascular disease in congenital heart disease. *Heart and Vessels*.

[11] Miyamoto S, Nagaya N, Satoh T, Kyotani S, Sakamaki F, Fujita M (2000). Clinical correlates and prognostic significance of six-minute walk test in patients with primary pulmonary hypertension. Comparison with cardiopulmonary exercise testing. *American Journal of Respiratory and Critical Care Medicine*.

[12] Ambrosy AP, Vaduganathan M, Huffman MD, Khan S, Kwasny MJ, Fought AJ (2012). Clinical course and predictive value of liver function tests in patients hospitalized for worsening heart failure with reduced ejection fraction: an analysis of the EVEREST trial. *European Journal of Heart Failure*.

[13] Takeda Y, Takeda Y, Tomimoto S, Tani T, Narita H, Kimura G (2010). Bilirubin as a prognostic marker in patients with pulmonary arterial hypertension. *BMC Pulmonary Medicine*.

[14] Simonneau G, Robbins IM, Beghetti M, Channick RN, Delcroix M, Denton CP (2009). Updated clinical classification of pulmonary hypertension. *Journal of the American College of Cardiology*.

[15] Al-Bawardy R, Vemulapalli S, Thourani VH, Mack M, Dai D, Stebbins A (2020). Association of Pulmonary Hypertension With Clinical Outcomes of Transcatheter Mitral Valve Repair. *JAMA Cardiology*.

[16] Samsky MD, Patel CB, DeWald TA, Smith AD, Felker GM, Rogers JG (2013). Cardiohepatic interactions in heart failure: an overview and clinical implications. *Journal of the American College of Cardiology*.

[17] van Deursen VM, Damman K, Hillege HL, van Beek AP, van Veldhuisen DJ, Voors AA (2010). Abnormal liver function in relation to hemodynamic profile in heart failure patients. *Journal of Cardiac Failure*.

[18] Giallourakis CC, Rosenberg PM, Friedman LS (2002). The liver in heart failure. *Clinics in Liver Disease*.

[19] Lau GT, Tan HC, Kritharides L (2002). Type of liver dysfunction in heart failure and its relation to the severity of tricuspid regurgitation. *The American Journal of Cardiology*.

[20] Li Y, Sheng H, Yan Z, Guan B, Qiang S, Qian J (2022). Bilirubin stabilizes the mitochondrial membranes during NLRP3 inflammasome activation. *Biochemical Pharmacology*.

[21] Song S, Wang S, Ma J, Yao L, Xing H, Zhang L (2013). Biliverdin reductase/bilirubin mediates the anti-apoptotic effect of hypoxia in pulmonary arterial smooth muscle cells through ERK1/2 pathway. *Experimental Cell Research*.

[22] Xu D, Hu YH, Gou X, Li FY, Yang XYC, Li YM (2022). Oxidative Stress and Antioxidative Therapy in Pulmonary Arterial Hypertension. *Molecules*.

[23] Hennigs JK, Cao A, Li CG, Shi M, Mienert J, Miyagawa K (2021). PPARγ-p53-Mediated Vasculoregenerative Program to Reverse Pulmonary Hypertension. *Circulation Research*.

[24] Reis GS, Augusto VS, Silveira APC, Jordão AA, Baddini-Martinez J, Poli Neto O (2013). Oxidative-stress biomarkers in patients with pulmonary hypertension. *Pulmonary Circulation*.

[25] Curjuric I, Imboden M, Adam M, Bettschart RW, Gerbase MW, Künzli N (2014). Serum bilirubin is associated with lung function in a Swiss general population sample. *The European Respiratory Journal*.

[26] Xu XQ, Lv ZC, Liu QQ, Zhao QH, Wu Y, Sun K (2017). Direct bilirubin: A new risk factor of adverse outcome in idiopathic pulmonary arterial hypertension. *International Journal of Cardiology*.

[27] Sun LJ, Zhang FC, Li D, Chen FR, Cui M, Gao W (2012). The impact of serum total bilirubin level on long-term prognosis in patients with chronic heart failure. *Zhonghua Nei Ke Za Zhi*.

[28] Gong JN, Zhai ZG, Yang YH, Liu Y, Gu S, Kuang TG (2015). Serum Bilirubin and 6-min Walk Distance as Prognostic Predictors for Inoperable Chronic Thromboembolic Pulmonary Hypertension: A Prospective Cohort Study. *Chinese Medical Journal*.

[29] Yan L, Huang Z, Zhao Z, Zhao Q, Tang Y, Zhang Y (2022). The Prognostic Impact of Serum Uric Acid on Disease Severity and 5-Year Mortality in Patients With Idiopathic Pulmonary Artery Hypertension. *Frontiers in Medicine*.

[30] Savale L, Akagi S, Tu L, Cumont A, Thuillet R, Phan C (2021). Serum and pulmonary uric acid in pulmonary arterial hypertension. *The European Respiratory Journal*.

