# Supplementary material for: Noninvasive Monitoring of Severe Pulmonary Artery Hypertension in Atrial Septal Defect Patients: Role of Serum Bilirubin Combined with Uric Acid
Source: Rev Cardiovasc Med. 2024 Jan 29;25(2):50. doi: 10.31083/j.rcm2502050 (PMC11263178; doi:10.31083/j.rcm2502050)
Supplement: Supplementary file 1 [file 2153-8174-25-2-050-s1.pdf]

Reviews in Cardiovascular Medicine

Supplementary Table 1: Comparison of blood biomarkers in different ASD groups stratified by mean pulmonary arterial pressure levels.

Variables	Without PAH n=34	Mild PAH n=22	Moderate PAH n=18	Severe PAH n=28	*P* Value
**mPAP, mmHg**	15.2±4.1	26.2±4.5	38.9±3.2	50.4±4.3	<0.001
**DBIL, μmol/L**	2.1±1.1	1.9±1.3	3.6±1.7	5.2±3.0	<0.001
**TBIL, μmol/L**	8.5±3.7	9.0±3.9	14.4±5.1	24.6±20.6	<0.001
**DBIL/TBIL**	0.3±0.1	0.2±0.1	0.3±0.1	0.3±0.1	0.35
**ALT, U/L**	23.2±17.8	17.3±4.9	20.1±15.1	18.0±6.4	0.30
**AST, U/L**	19.8±6.8	19.5±3.9	19.2±5.8	20.5±9.1	0.91
**UA, μmol/L**	317.7±74.0	312.5±55.7	324.4±72.3	403.6±131.5	<0.001
**NT-proBNP, pg/ml**	52(30.5,110.3)	52.9(38.8,125)	60.9(21.6,144.9)	525(129.3,626)	<0.001

SPAH: severe pulmonary artery hypertension; DBIL: direct bilirubin; TBIL: total bilirubin. ALT: alanine aminotransferase; AST: aspartate aminotransferase; UA: uric acid.

Supplementary Table 2: Receiver operating characteristic curve analysis of blood biomarkers in predicting ASD patients suffering from SPAH.

Variables	Sensitivity	Specificity	AUC	95% CI	*P*
**DBIL**	92.9%	51.4%	0.794	(0.701, 0.866)	<0.001
**TBIL**	89.3%	62.2%	0.788	(0.685, 0.890)	<0.001
**NT-proBNP**	71.4%	87.8%	0.836	(0.748, 0.923)	<0.001
**UA**	50.0%	90.5%	0.693	(0.563, 0.824)	0.003
**DBIL-UA**	92.9%	71.6%	0.874	(0.799, 0.949)	<0.001

ASD: atrial septal defect; SPAH: severe pulmonary artery hypertension; DBIL: direct bilirubin; DBIL: total bilirubin. UA: uric acid.

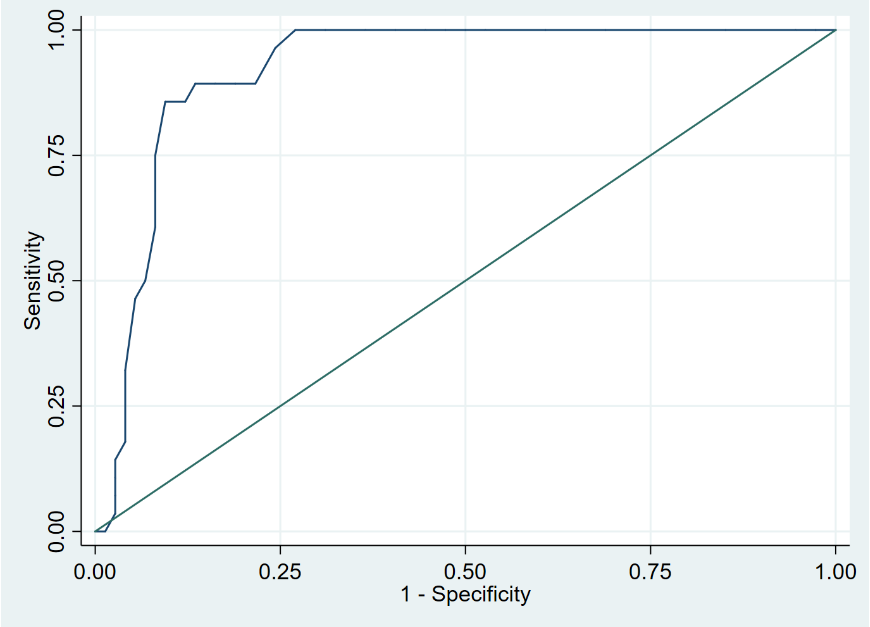

Supplementary Fig. 1: ROC analyses of RVD predicting ASD patients with SPAH. ROC: receiver operating characteristic; ASD: atrial septal defect; SPAH: severe pulmonary artery hypertension.

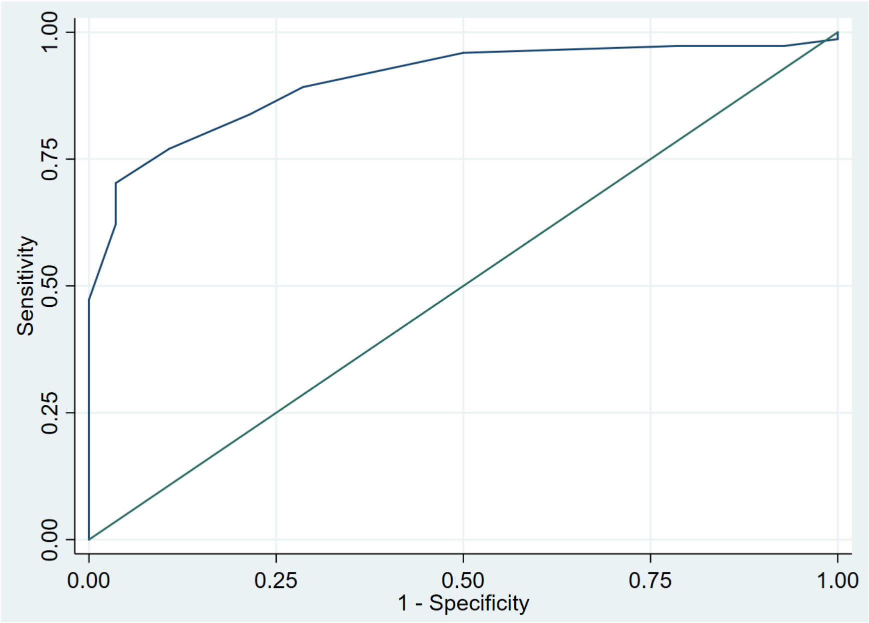

Supplementary Fig. 2: ROC analyses of TAPSE predicting ASD patients with SPAH. ROC: receiver operating characteristic; ASD: atrial septal defect; SPAH: severe pulmonary artery hypertension.
